# Impaired macrophage and memory T-cell responses to Bacillus Calmette-Guerin nonpolar lipid extract

**DOI:** 10.3389/fimmu.2023.1263352

**Published:** 2024-01-11

**Authors:** Alice Sarno, Avelina Leite, Carlos Augusto, Igor Muller, Luanna de Ângelis, Lilian Pimentel, Adriano Queiroz, Sergio Arruda

**Affiliations:** ^1^Advanced Laboratory of Public Health, Gonçalo Moniz Institute, Fiocruz, Salvador, Brazil; ^2^Department of Pathology and Forensic Medicine, Faculty of Medicine, Federal University of Bahia, Salvador, Brazil; ^3^Laboratory of Immunoepidemiology, Aggeu Magalhães Institute, Fiocruz, Recife, Brazil; ^4^Department of Life Sciences, State University of Bahia, Salvador, Brazil

**Keywords:** Bacillus Calmette-Guerin, *Mycobacterium tuberculosis*, nonpolar lipid extracts, macrophage gene expression, memory T-cell responses

## Abstract

**Introduction:**

The attenuation of BCG has led to the loss of not only immunogenic proteins but also lipid antigens.

**Methods:**

Thus, we compared the macrophage and T-cell responses to nonpolar lipid extracts harvested from BCG and *Mycobacterium tuberculosis* (*Mtb*) to better understand the role of BCG lipids in the already known diminished responses of the vaccine strain.

**Results:**

Relative to Mtb, nonpolar lipid extract from BCG presented a reduced capacity to trigger the expression of the genes encoding TNF, IL-1b, IL-6 and IL-10 in RAW 264.7 macrophages. Immunophenotyping of PBMCs isolated from healthy individuals revealed that lipids from both BCG and *Mtb* were able to induce an increased frequency of CD4^+^ and CD8^+^ T cells, but only the lipid extract from *Mtb* enhanced the frequency of CD4^-^CD8^-^double-negative, γσ^+^, CD4^+^HLA-DR^+^, and γσ^+^HLA-DR^+^ T cells relative to the nonstimulated control. Interestingly, only the *Mtb* lipid extract was able to increase the frequency of CD4^+^ memory (CD45RO^+^) T cells, whereas the BCG lipid extract induced a diminished frequency of CD4^+^ central memory (CD45RO^+^CCR7^-^) T cells after 48 h of culture compared to *Mtb*.

**Discussion:**

These findings show that the nonpolar lipids of the BCG bacilli presented diminished ability to trigger both proinflammatory and memory responses and suggest a potential use of *Mtb* lipids as adjuvants to increase the BCG vaccine efficacy.

## 1 Introduction

Tuberculosis (TB), caused by *Mycobacterium tuberculosis* (*Mtb*), is one of the leading infectious diseases worldwide, with 10.6 million new cases and 1.6 million deaths in 2021 (1). BCG (Bacillus Calmette-Guérin) is currently the only licensed vaccine against pulmonary TB, despite its variable efficacy (0-80%) (2–4).

Composed of attenuated *M. bovis* bacilli, BCG has accumulated genomic polymorphisms that account for the absence of not only protein antigens but also key lipid antigens (5–9). A lipidomic analysis compared the lipid profiles of *Mtb* and BCG and revealed more than 1,000 differences between both strains (7). Recently, we performed an *in silico* evaluation and found 14 nonhomologous lipid-related genes absent in the six BCG strains most used worldwide relative to *Mtb*. Those genes were associated with the functional categories “cell wall and cell processes”, “virulence, detoxification and adaptation”, “lipid metabolism”, and “intermediary metabolism and respiration”, and together, these gene modifications may favor a dormant-like state of the BCG strains (10).

Mycobacterial lipids play a crucial role in the immunopathogenesis of TB (11). Petrilli et al. (2020) showed differential macrophage and T-cell responses to lipids extracted from two *Mtb* strains, an ATP-binding cassette transporter-knockout strain and its parental strain, highlighting the role of these molecules in controlling the inflammatory response (12). In BCG, the absence of lipid antigens has been associated with important changes in the host’s immune response, with consequent decreased control of mycobacterial burden and vaccine protection *in vivo* (13–15).

Protection against TB relies on the induction of a strong cellular immune response, although correlates of protection have not been identified. The results from a phase IIb clinical trial with the candidate MVA85A did not add protection against TB, despite significant induction of T-helper type 1 (Th1) cells (16, 17). Only recent clinical trial results have shown that it is possible to boost the protection already conferred by BCG throughout the revaccination of adolescents (18) and by the immunization of adults with the candidate M72/ASO1E (19). Other promising candidates have been proposed, including relevant findings from nonhuman primate models, that have been shown to induce not only CD4 and CD8 T cells but also polyfunctional Th17 cells and interleukin-10 production (20, 21). However, these results have not yet reached public health action.

The role of protein antigens has already been comprehensively described, whereas the importance of lipid antigens in the host’s immune response has been less explored. Here, we aimed to compare the cellular immune response induced by BCG and *Mtb* lipid extracts to better understand the influence of lipid losses on strain attenuation. These findings could elucidate future studies on the use of this class of antigens in new vaccine candidates to promote a more effective response and protection in combination with proteins.

## 2 Materials and methods

### 2.1 Bacterial strains, growth conditions, and lipid extraction

*M. bovis* BCG Moreau (BCG Moreau RDJ, FAP) and M. tuberculosis Erdman strains were used. Both strains were cultured in Middlebrook 7H9 broth (Difco, MD) supplemented with 10% ADC (Beckton-Dickinson, MD) and incubated at 37°C and 5% CO_2_ until stationary phase. Then, planktonic cultures of BCG Moreau and *Mtb* were harvested and used for extraction of nonpolar lipids (22, 23). Briefly, 5 mL of methanol with 0.3% NaCl (100:10) and 2.5 mL of petroleum ether were added to 30 mL of cultures and incubated for 30 min at room temperature. The upper petroleum ether layer containing the nonpolar lipids was collected after centrifugation and kept in glass flasks until complete solvent evaporation. Nonpolar lipid extracts of each strain were weighed and resuspended in hexane:isopropanol (1:1) at 0.02 mg/mL. The nonpolar fraction is expected to have phthiocerol dimycocerosates (PDIM), triacylglycerol (TAG), pentacyl trehalose (PAT), trehalose monomycolate (TMM), and dimycolate (TDM, the cord factor), among others (24). Finally, 24-well tissue culture plates were layered with 0.5 mL of lipid extracts or hexane:isopropanol. Solvent evaporation was allowed, and plates were kept at -20°C until use.

### 2.2 RAW macrophage assay

#### 2.2.1 RAW 264.7 murine macrophage culture

RAW 264.7 cells (ATCC TIB-71) were cultured in Dulbecco’s modified Eagle’s medium (DMEM; Gibco) supplemented with 10% FBS at 37°C and 5% CO_2_. After achieving 70% confluency, macrophages were seeded onto lipid-coated 24-well tissue culture plates at 3.7×10^5 cells/well and incubated at 37°C and 5% CO_2_ for 2 h, 12 h, 24 h or 72 h. For the control samples, wells were coated with hexane/isopropanol in the absence of lipid extracts. Staining with trypan blue (Gibco) was used to assess cell number and viability.

#### 2.2.2 RNA extraction and purification

Total RNA was extracted from RAW cells using the TRIzol RNA extraction protocol (Invitrogen, Life Technologies) and treated with DNase (Qiagen). DNA-free RNA (500 ng) was mixed with 50 ng of random hexamers and 50 μM oligo (dT) (Invitrogen), and cDNA was synthesized by Superscript III reverse transcriptase (Invitrogen) following the manufacturer’s recommendations.

#### 2.2.3 RT-qPCR

The expression of the TNFα, IL-1β, IL-6 and IL-10 genes was measured (**Supplementary Table 1**). Primers were designed to produce a 100–195 bp amplicon for each gene. qPCRs were performed using 25 ng of cDNA and Maxima SYBR Green/ROX qPCR Master Mix (2X) (Thermo Fisher) following the manufacturer’s recommendations. The expression levels of all target genes were normalized to β-actin and glyceraldehyde 3-phosphate dehydrogenase (GAPDH), and relative changes between lipid-stimulated and nonstimulated RAW cells were measured by 2-ΔΔCt (25).

### 2.3 Assays with peripheral blood mononuclear cells

#### 2.3.1 Study participants

Participants (n = 12) were enrolled in this study and recruited from Gonçalo Moniz Institute (FIOCRUZ). All participants had been vaccinated with BCG during infancy in accordance with national guidelines and tested negative for latent TB infection by interferon-γ release assay (QuantiFERON® TB Gold Plus) upon enrollment. The mean age of the volunteers was 28.08 years (SD = 6.37) with 66.7% identified as female. Information about previous contact with TB patients and HIV status was self-declared, adhering regulation from the Research Ethics Committee at Gonçalo Moniz Institute (FIOCRUZ) (approved protocol number: 57273322.4.0000.0040). Individuals with regular contact with TB patients, those who reported recent episodes of cough and/or fever, or those with positive or indeterminate interferon-γ release assay results were excluded from the study.

#### 2.3.2 PBMC isolation and culture assays

Peripheral blood mononuclear cells (PBMCs) were obtained by Ficoll-Paque (GE Healthcare) density gradient and cryopreserved in liquid nitrogen with inactive fetal bovine serum (FBS) and 10% DMSO before culture assays. Cryopreserved cells were then thawed, and PBMC concentrations were adjusted to 106 cells/mL in 1 mL of RPMI 1640 (with 2 mM L-glutamine and 30 mM HEPES) containing 1% gentamicin and 10% FBS (GIBCO). PBMCs were added to 24-well tissue culture plates previously prepared with nonpolar lipid extracts from BCG and *Mtb*. Phytohemagglutinin (PHA) (GIBCO) (10 μg/mL) was added as a positive control. Cells were cultured for 24 h, 48 h and 72 h at 37°C in a 5% CO_2_ humidified atmosphere, and the 48 h time-point was chosen for the analyses.

#### 2.3.3 Flow cytometry and cytokine analyses

Cells were first stained with CD3-FITC, CD4-PE, CD8-APC-Cy7, CD45RA PE-Cy7, CD45RO-APC, CCR7-BV510, HLA-DR-BV605, and TCRγδ-BV421 (BD Biosciences). For intracellular staining with IFNγ-PE-Cy7, TNF-AL700, IL-2-BV421, and IL-17-BV510, cells were fixed and permeabilized using the BD Biosciences Cytofix/Cytoperm Kit. Data were acquired on BD LSRFortessa® (50,000 events), and cell frequencies, as well as median fluorescence intensity (MFI), were measured using FlowJo 10 software (Tree Star Inc.). Supernatants of PBMC cultures were collected and stored at -20°C for cytokine assays. Concentrations of IFNγ and IL-10 were measured by ELISA (R&D Systems) according to the manufacturer’s instructions.

#### 2.3.4 Statistical analyses

Statistical analyses were performed using GraphPad Prism 8 software (GraphPad Inc.). Normal distribution was assessed by the Shapiro−Wilk test. Statistical significance was assessed by Student’s t test, one-way ANOVA followed by Tukey’s posttest, or Kruskal−Wallis followed by Dunn’s posttest. The results were considered significant when p <0.05.

## 3 Results

### 3.1 Lipid extract from the BCG strain induced lower expression levels of proinflammatory genes relative to *Mtb* lipids

The transcriptional expression of genes encoding pro- and anti-inflammatory cytokines was measured in macrophages cultured with nonpolar lipid extracts harvested from both BCG and *Mtb* strains.

**Figure 1:**
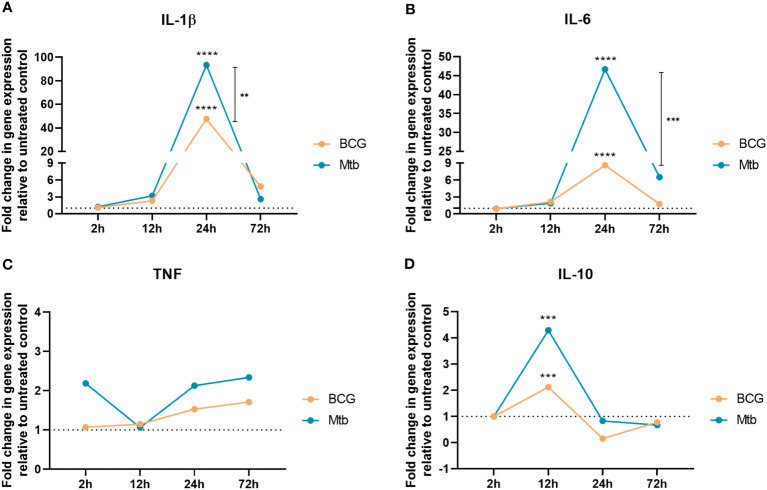
RT‒qPCR analyses of **(A)** *IL-1β*, **(B)** *IL-6*, **(C)** *TNF*, and **(D)** *IL-10* after 2 h, 12 h, 24 h, and 72 h of cell exposure to BCG (orange) and Mtb (blue) lipid extracts. Data represent the mean fold-change difference between BCG and Mtb relative to untreated control. Gene expression was normalized to *glyceraldehyde 3-phosphate dehydrogenase* (GAPDH) and *β -actin * genes. *p* values were calculated by t test, with ***p <0.001; ****p <0.0001.

Lipids from the BCG strain induced lower transcript production than *Mtb* for all evaluated genes at most time points (**Figure 1**). Relative to the nonstimulated control, there was increased expression of IL-1β and IL-6 at 24 h of culture in macrophages cultured with both BCG and *Mtb* lipid extracts (p <0.0001) (**Figures 1A, B**). The expression of IL-1β and IL-6 increased by 48- and 9-fold in macrophages stimulated with BCG lipid extract and by 93- and 47-fold in *Mtb* lipid-induced cells, respectively (**Supplementary Table 2**). In addition, BCG lipids induced lower expression of TNF across the 2 h, 24 h and 72 h time points when compared with the stimulus triggered by *Mtb* lipids (**Figure 1C**). Whereas *Mtb* lipids sustained a 2-fold upregulation of this gene, the 2-ΔΔCt of TNF in BCG lipid-stimulated macrophages varied from 1.1 to 1.7 over the 2 h, 24 h and 72 h time points (**Supplementary Table 2**). After 12 h of incubation, the expression of IL-10 was 2- and 4-fold in cultures with lipids extracted from BCG and *Mtb*, respectively (p <0.001) (**Figure 1D**).

### 3.2 Both BCG and *Mtb* nonpolar lipid extracts increased the frequency of CD4+ and CD8+ T cells

**Figure 2:**
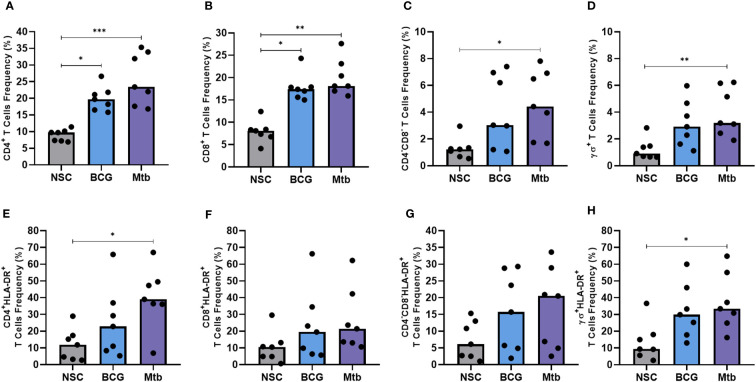
Flow cytometry of conventional and nonconventional T cells after 48 h of *in vitro* culture of PBMCs from healthy individuals with lipid extracts of BCG and Mtb. **(A)** and **(E)** Frequencies of CD4^+^ and CD4^+^ HLA-DR^+^ T cells. **(B)** and **(F)** Frequencies of CD8^+^ and CD8^+^ HLA-DR^+^ T cells. **(C)** and **(G)** Frequencies of CD4^-^CD8^-^ DN and CD4^-^CD8^-^ DN HLA-DR^+^ T cells. **(D)** and **(H)** Frequencies of γδ^+^ and γδ^+^ HLA-DR^+^ T cells. Normal distribution was determined by the Shapiro‒Wilk test. *p values* for normal distributions were calculated by one-way ANOVA, and *p values* for nonnormal distributions were calculated by the Kruskal‒Wallis test. *p <0.05; **p <0.01; ***p <0.001. Nonstimulated control (negative control); Phytohemagglutinin, PHA (positive control).

To evaluate whether lipids from BCG could also elicit a lymphocyte response, PBMCs obtained from healthy individuals were cultured with lipid extracts and stained for immunophenotyping (**Supplemetary Figure 1**). Relative to nonstimulated controls, lipids from both strains enhanced the frequencies of CD4+ and CD8+ T cells (p <0.05) (**Figures 2A, B**), whereas only *Mtb* significantly increased the frequencies of CD4-CD8- double-negative (DN) and γδ+ T cells (p <0.05 and p <0.01, respectively) (**Figures 2C, D**). Similarly, *Mtb* but not BCG lipids induced the proliferation of HLA-DR-positive CD4+ and γδ+ T cells (p <0.05) (**Figures 2E, H**).

### 3.3 BCG lipids present a reduced capacity to induce CD4+ memory and central memory T-cell proliferation

**Figure 3:**
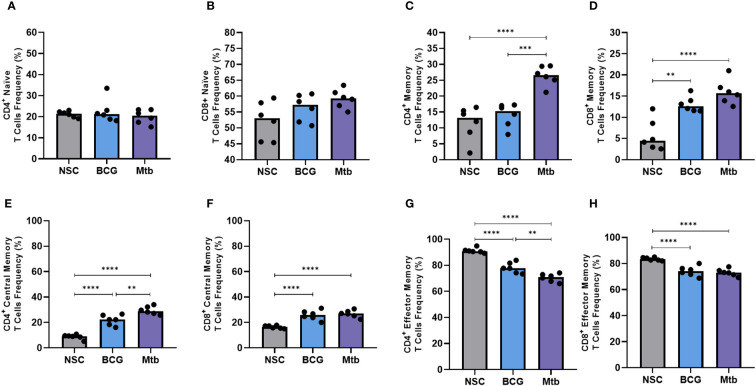
Flow cytometry of memory T cells after 48 h of *in vitro* culture of PBMCs from healthy individuals with lipid extracts of BCG and Mtb. **(A)** and **(B)** Frequencies of CD4^+^ and CD8^+^ naïve T cells (CD45RA^+^). **(C)** and **(D)** Frequencies of CD4^+^ and CD8^+^ memory T cells (CD45RO^+^). **(E)** and **(F)** Frequencies of CD4^+^ and CD8^+^ central memory T cells (CD45RO^+^ CCR7^+^). **(G)** and **(H)** Frequencies of CD4^+^ and CD8^+^ effector memory T cells (CD45RO^+^ CCR7^-^). Normal distribution was determined by the Shapiro‒Wilk test. *p values* for normal distributions were calculated by one-way ANOVA, and p values for nonnormal distributions were calculated by the Kruskal‒Wallis test. **p <0.01; ***p <0.001; ****p <0.0001. Nonstimulated control (negative control); Phytohemagglutinin, PHA (positive control).

Neither BCG nor *Mtb* lipid extract stimulation resulted in significant changes in the frequencies of CD4+ and CD8+ naïve T cells (**Figures 3A, B**). Conversely, *Mtb* lipids induced both CD4+ and CD8+ memory T cells (p <0.0001), whereas lipid extract from BCG only increased the frequencies of the latter (p <0.01) when compared to the nonstimulated controls (**Figures 3C, D**). BCG lipids were not only unable to increase the frequency of CD4+ memory T cells but also induced significantly lower proliferation of this population than *Mtb* lipids (p <0.001) (**Figure 3C**).

Nonpolar lipid extracts from both BCG and *Mtb* strains enhanced the frequency of CD4+ and CD8+ central memory T cells (p <0.0001) (**Figures 3E, F**) but induced lower proliferation of CD4+ and CD8+ effector memory T cells (p <0.0001) (**Figures 3G, H**) when compared to nonstimulated controls. Furthermore, compared to *Mtb*, BCG lipid extract induced significantly lower frequencies of central memory in CD4+ T-cell populations (p <0.01) (**Figure 3E**).

### 3.4 Distinct level of cytokine synthesis between *Mtb*- and BCG-lipid-induced T cells

**Figure 4:**
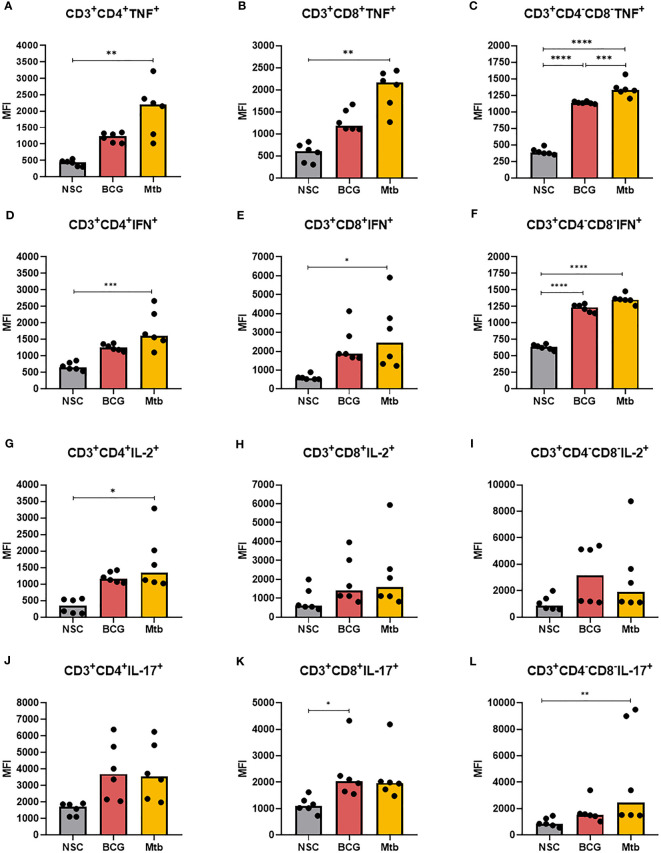
Flow cytometry of CD4^+^, CD8^+^ and CD4^-^CD8^-^ DN T cells producing TNF, IFNγ, IL-2, and IL-17 after 48 h of *in vitro* culture of PBMCs from healthy individuals with lipid extracts of BCG and Mtb. **(A)**, **(B)**, and **(C)** MFI of CD4^+^, CD8^+^ and CD4^-^CD8^-^ DN T cells producing TNF. **(D)**, **(E)**, and **(F)** MFI of CD4^+^, CD8^+^ and CD4^-^CD8^-^ DN T cells producing IFNγ. **(G)**, **(H)**, and **(I)** MFI of CD4^+^, CD8^+^ and CD4^-^CD8^-^ DN T cells producing IL-2. **(J)**, **(K)**, and **(L)** MFI of CD4^+^, CD8^+^ and CD4^-^CD8^-^ DN T cells producing IL-17. Normal distribution was determined by the Shapiro‒Wilk test. *p values* for normal distributions were calculated by one-way ANOVA, and *p* values for nonnormal distributions were calculated by the Kruskal‒Wallis test. *p <0.05; **p <0.01; ***p <0.001; ****p <0.0001. MFI: Median fluorescence intensity. Nonstimulated control (negative control); Phytohemagglutinin, PHA (positive control).

Intracellular cytokine levels in CD4+, CD8+, and CD4-CD8- DN T cells in lipid-induced PBMCs from healthy individuals were also assessed (**Figure 4**; **Supplementary Table 3**). Relative to nonstimulated cells, *Mtb* lipid extracts significantly increased the intracellular levels of TNF and IFNγ in all evaluated cell populations (**Figures 4A-F**). Lipids from *Mtb* also triggered the synthesis of IL-2 in CD4+ (**Figure 4G**) (p <0.05) and IL-17 in CD4-CD8- DN T cells (**Figure 4L**) (p <0.01). BCG lipid extract stimuli were able to only enhance the MFI of TNF and IFNγ+ in CD4-CD8- DN T cells (p <0.0001) (**Figures 4C, F**) and IL-17 in CD8+ T cells (**Figure 4K**) (p <0.05). Compared to *Mtb*, the BCG lipid extract induced significantly lower expression of TNF in CD4-CD8- DN cells (p <0.001) (**Figure 4F**).

**Figure 5:**
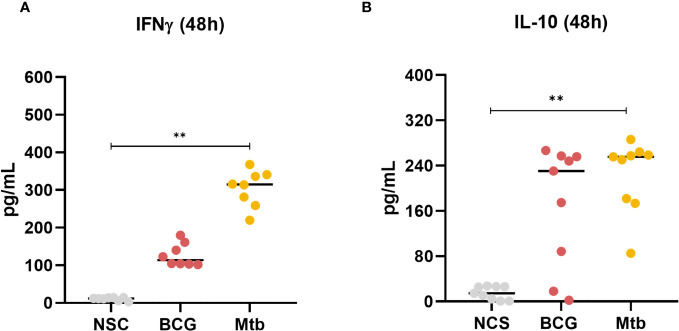
Concentrations of **(A)** IFNγ and **(B)** IL-10 production after 48 h of *in vitro* culture of PBMCs from healthy individuals with lipid extracts of BCG and Mtb. Normal distribution was determined by the Shapiro‒Wilk test. *p values* for normal distributions were calculated by one-way ANOVA, and *p* values for nonnormal distributions were calculated by the Kruskal‒Wallis test. **p <0.01. Nonstimulated control (negative control); Phytohemagglutinin, PHA (positive control).

The concentrations of IFNγ and IL-10 in culture supernatants were measured at 48 h. Only *Mtb* lipid extract increased the concentration of both cytokines (p <0.01) (**Figures 5A, B**; **Supplementary Table 5**).

## 4 Discussion

Consecutive *in vitro* passages of *M. bovis* gave rise to BCG, an attenuated strain with depletion of at least nine regions of difference (RD), including RD1, which encodes the proteins from the ESX-1 secretion system ESX-1, such as ESAT-6 and CFP-10 (9). Although these proteins are important virulence factors, their deletion does not completely explain the reduced ability of BCG to induce a protective immune response after vaccination (14, 15, 26). The lipid profiles of *Mtb* and BCG differ from each other in more than 1,000 species (7), whereas an *in silico* analysis showed that lipid-related gene deletions hinder the production of mce3, enoyl-CoA hydratase, and phospholipase C in BCG. Thus, we reasoned that losses of nonpolar lipid antigens in BCG would contribute to impairing host cell functions and might determine whether the vaccine can be improved by adding *Mtb*-derived lipid adjuvants.

Compared to *Mtb*, we observed that BCG lipids predominantly induced diminished macrophage expression of the pro-inflammatory markers IL-1β, IL-6, and TNF. This diminished ability of BCG nonpolar lipids to induce inflammatory responses was also evidenced by the synthesis of TNF and IFNγ in both CD4+ and CD8+ T cells. Conversely, the difference between each stimulus was less evident in TNF- and IFNγ-producing CD4-CD8- DN T cells. Considering that the frequencies of these populations are relatively low compared to conventional T cells, we can assume that the BCG strain also has an impaired ability to induce inflammation at the adaptive immune response level.

Lipid extracts from *Mtb* and BCG strains were able to, directly or indirectly, activate several T-cell subpopulations, according to the analyses performed here. Interestingly, there was no difference in the frequencies of CD4+ and CD8+ T cells in cultures with both lipid extracts. In addition, although there was a significant increase in the frequencies of CD4-CD8- DN and γδ+ T cells in cultures with *Mtb* lipid extract, relative to the untreated control, there were no distinguishable differences in the proportions of these cells between cultures with BCG and *Mtb* lipid extracts. These data suggest that despite the reduced capacity of BCG nonpolar lipids to induce inflammation, depletions in the genes related to lipid metabolism did not alter the ability of this strain to increase the frequency of some subsets of T cells.

Furthermore, different TB vaccine candidates and inoculation routes have been associated with the activation of distinct memory T-cell subsets, but there is no consensus about which cell subtype is responsible for providing protection. Here, *Mtb* lipid extracts induced a greater response from memory T cells than BCG, especially CD4+ T cells, which have been associated with vaccine candidates that use lipid formulations (27, 28). In addition, lipid extracts from both BCG and *Mtb* strains prompted higher and lower frequencies of effector and central memory T cells, respectively. In particular, effector memory cells are induced by BCG vaccination in humans and represent the predominant cell population in the lungs of vaccinated mice, which has been associated with stronger and more efficient protection against infection (29–32). Conversely, smaller populations of central memory T cells result in poorer memory responses (33–36). Notably, *Mtb* lipids were associated with a greater increase of central memory and decrease of effector memory T cells, whereas BCG lipids induced a similar yet diminished dynamic between these populations. This finding might indicate that *Mtb* lipids have a better chance to induce a long-lasting T-cell memory response than BCG lipids.

This study incorporated a wide range of lymphocyte and cytokine markers to compare the cellular immune response induced by total nonpolar lipid extracts from *Mtb* and BCG. To the best of our knowledge, no other study has performed T-cell immunophenotyping under such stimulation. To minimize potential biases arising from current or previous *Mtb* infections, only healthy and IGRA-negative participants were included in this study. However, this approach precluded us from investigating the responses of cells pre-activated by Mtb to both *Mtb* and BCG lipids.

Notably, measuring the levels of CD1a receptors would increase our comprehension of the abilities of *Mtb* and BCG nonpolar lipids in stimulating CD4+ and CD8+ T cells. Additionally, subsequent evaluations of different antigen delivery models that more accurately simulate bacteria-cell interactions and ex vivo experiments are important to assess whether the lipid-induced immune response contributes to bacterial clearance.

The question that arises from these analyses is whether the nonpolar lipids from *Mtb* could in fact induce long lasting memory during a BCG vaccination and, if that is the case, which lipid species are responsible for such activation. Although our study could not provide evidence of long-term protection induced by mycobacterial lipids, our results support the possibility of improvement of the BCG vaccine by including *Mtb* lipid molecules as adjuvants in the vaccination scheme against TB.

## Data availability statement

The original contributions presented in the study are included in the article/**Supplementary Material**. Further inquiries can be directed to the corresponding author.

## Ethics statement

The studies involving humans were approved by Research Ethics Committee at Gonçalo Moniz Institute. The studies were conducted in accordance with the local legislation and institutional requirements. The participants provided their written informed consent to participate in this study.

## Author contributions

AS: Conceptualization, Formal analysis, Investigation, Writing – original draft. AL: Investigation, Writing – review & editing. CA: Investigation, Writing – review & editing. IM: Investigation, Writing – review & editing. LA: Investigation, Writing – review & editing. LP: Supervision, Writing – review & editing. AQ: Conceptualization, Formal analysis, Project administration, Supervision, Writing – original draft. SA: Conceptualization, Project administration, Supervision, Writing – review & editing.
