# Supplementary figures and images for: Impaired macrophage and memory T-cell responses to Bacillus Calmette-Guerin nonpolar lipid extract
Source: Front Immunol. 2024 Jan 11;14:1263352. doi: 10.3389/fimmu.2023.1263352 (PMC10808680; doi:10.3389/fimmu.2023.1263352)

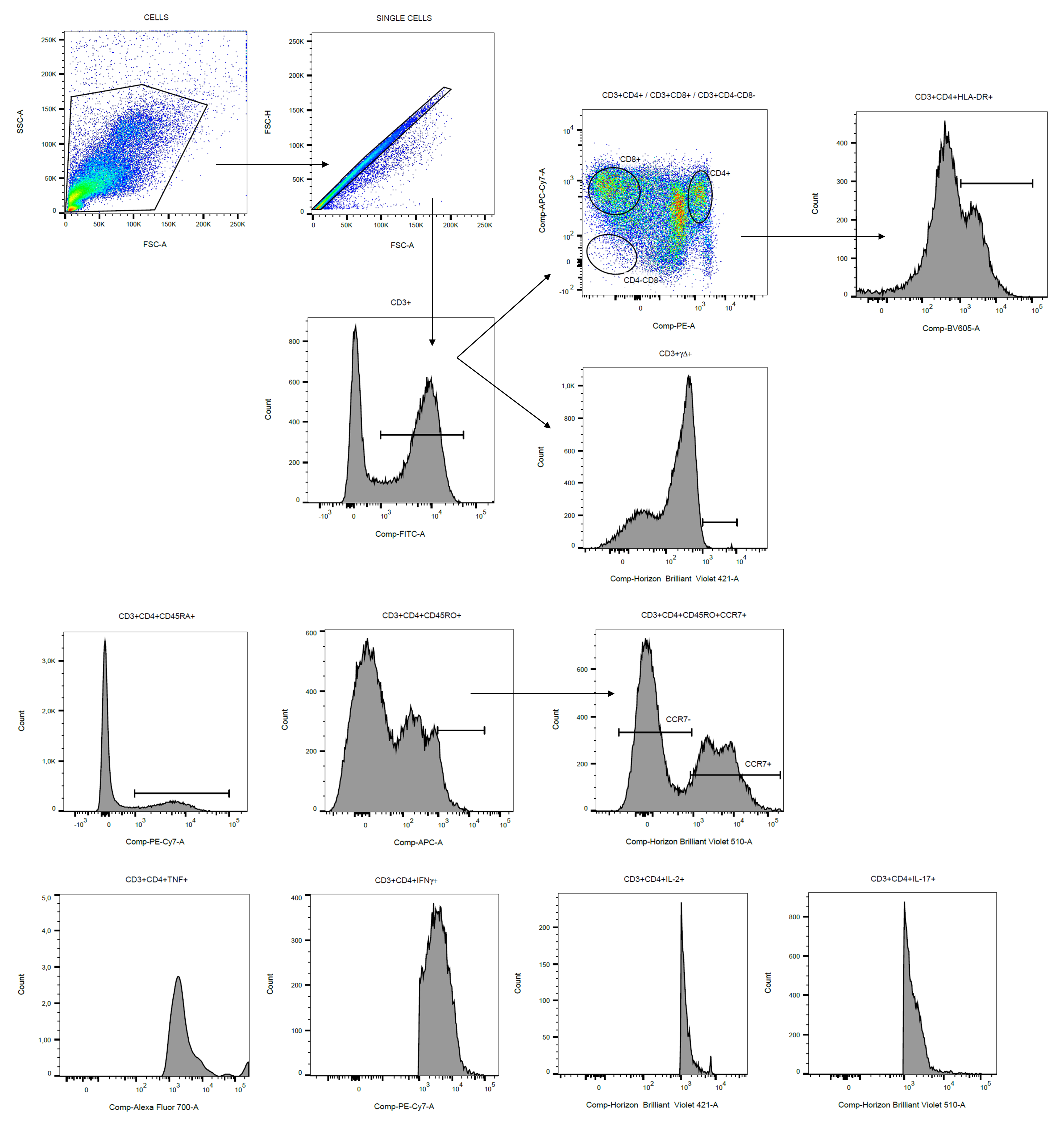

Supplement: Supplementary file 1 [file Image_1.tif]
