# Supplementary material for: Impaired macrophage and memory T-cell responses to Bacillus Calmette-Guerin nonpolar lipid extract
Source: Front Immunol. 2024 Jan 11;14:1263352. doi: 10.3389/fimmu.2023.1263352 (PMC10808680; doi:10.3389/fimmu.2023.1263352)
Supplement: Supplementary file 2 [file Table_1.docx]

**Supplementary Table 1.** List of primers used for RT-qPCR.

| **Gene** | **Nucleotide Sequence (5'3')** |
| --- | --- |
| TNF Forward | CTACCTTGTTGCCTCCTCTTT |
| TNF Reverse | GAGCAGAGGTTCAGTGATGTAG |
| IL-1β Forward | TCGTGCTGTCGGACCCATAT |
| IL-1β Reverse | GTCGTTGCTTGGTTCTCCTTGT |
| IL-6 Forward | CGAGAGTCCTTCAGAGAGATACA |
| IL-6 Reverse | CCTTCTGTGACTCCAGCTTATC |
| IL-10 Forward | CTGTAAAACAAGAGCAAGGC |
| IL-10 Reverse | GAAGCTTCTGTTGGCTCCC |
| GAPDH Forward | TCAACGGCACAGTCAAGG |
| GAPDH Reverse | ACTCCACGACATACTCAGC |
| β-actina Forward | GAGGTATCCTGACCCTGAAGTA |
| β-actina Reverse | CACACGCAGCTCATTGTAGA |
