# Supplementary material for: Impaired macrophage and memory T-cell responses to Bacillus Calmette-Guerin nonpolar lipid extract
Source: Front Immunol. 2024 Jan 11;14:1263352. doi: 10.3389/fimmu.2023.1263352 (PMC10808680; doi:10.3389/fimmu.2023.1263352)
Supplement: Supplementary file 3 [file Table_2.docx]

**Supplementary Table 2.** Mean, upper limit and lower limit of 2^-∆∆Ct^ values from RT-qPCR analyses.

|  | | **2h** | | | **12h** | | | **24h** | | | **72h** | | |
| --- | --- | --- | --- | --- | --- | --- | --- | --- | --- | --- | --- | --- | --- |
|  |  | **Mean** | **Upper Limit** | **Lower Limit** | **Mean** | **Upper Limit** | **Lower Limit** | **Mean** | **Upper Limit** | **Lower Limit** | **Mean** | **Upper Limit** | **Lower Limit** |
| **BCG** | **TNF** | 1.07 | 1.32 | 0.86 | 1.15 | 1.32 | 1.01 | 1.53 | 1.89 | 1.25 | 1.71 | 3.74 | 0.78 |
|  | **IL-1** | 1.10 | 1.26 | 0.96 | 2.31 | 2.87 | 1.85 | 47.67 | 51.99 | 43.71 | 4.86 | 7.40 | 3.19 |
|  | **IL-6** | 0.91 | 1.01 | 0.82 | 2.14 | 2.67 | 1.71 | 8.64 | 10.37 | 7.19 | 1.74 | 2.47 | 1.23 |
|  | **IL-10** | 1.00 | 1.00 | 1.00 | 2.12 | 4.26 | 1.06 | 0.16 | 0.26 | 0.10 | 0.78 | 2.02 | 0.30 |
| **Mtb** | **TNF** | 2.19 | 2.69 | 1.78 | 1.06 | 1.20 | 0.93 | 2.13 | 2.74 | 1.65 | 2.34 | 2.39 | 2.29 |
|  | **IL-1** | 1.23 | 1.53 | 0.99 | 3.19 | 3.93 | 2.60 | 93.37 | 106.88 | 81.58 | 2.61 | 2.65 | 2.57 |
|  | **IL-6** | 0.93 | 1.12 | 0.77 | 1.88 | 2.68 | 1.32 | 46.70 | 53.87 | 40.48 | 6.51 | 10.04 | 4.23 |
|  | **IL-10** | 1.00 | 1.00 | 1.00 | 4.29 | 7.96 | 2.31 | 0.83 | 1.26 | 0.55 | 0.67 | 0.86 | 0.52 |
