# Supplementary material for: Impaired macrophage and memory T-cell responses to Bacillus Calmette-Guerin nonpolar lipid extract
Source: Front Immunol. 2024 Jan 11;14:1263352. doi: 10.3389/fimmu.2023.1263352 (PMC10808680; doi:10.3389/fimmu.2023.1263352)
Supplement: Supplementary file 4 [file Table_3.docx]

**Supplementary Table 3.** Frequency values from Flow Cytometry analyses.

| **T CD4+** | | | | | **T CD8+** | | | | | **T CD4-CD8-** | | | | | **T γδ+** | | | | |
| --- | --- | --- | --- | --- | --- | --- | --- | --- | --- | --- | --- | --- | --- | --- | --- | --- | --- | --- | --- |
|  | **CN** | **BCG** | **Mtb** | **PHA** |  | **CN** | **BCG** | **Mtb** | **PHA** |  | **CN** | **BCG** | **Mtb** | **PHA** |  | **CN** | **BCG** | **Mtb** | **PHA** |
| **A07** | 9.7 | 21.8 | 23.4 | x | **A07** | 12.4 | 24.3 | 27.6 | x | **A07** | 2.955 | 6.95 | 6.91 | x | **A07** | 2.825 | 5.97 | 6.23 | x |
| **A01** | 9.75 | 19.7 | 17.6 | x | **A01** | 8.3 | 17.8 | 20.4 | x | **A01** | 1.34 | 3.03 | 3.95 | x | **A01** | 0.915 | 2.91 | 3.11 | x |
| **A02** | 6.90 | 15.8 | 31.90 | x | **A02** | 7.35 | 15.0 | 18.10 | x | **A02** | 0.68 | 1.1 | 1.74 | x | **A02** | 0.66 | 1.1 | 1.90 | x |
| **A04** | 7.2 | 16.5 | 16.8 | x | **A04** | 8.1 | 17.9 | 18.1 | x | **A04** | 0.54 | 1.2 | 1.68 | x | **A04** | 0.645 | 2.29 | 3.19 | x |
| **A06** | 7.35 | 18.2 | 22 | 61.1 | **A06** | 6.75 | 17.4 | 17 | 42.7 | **A06** | 1.10 | 2.98 | 4.42 | 9.7 | **A06** | 1.47 | 3.71 | 5.15 | 15.6 |
| **A08** | 10.15 | 26.6 | 35.3 | 57.90 | **A08** | 8.4 | 17 | 23.1 | 47.90 | **A08** | 1.23 | 6.2 | 6.49 | 13 | **A08** | 1.385 | 4.67 | 6.16 | 12.7 |
| **A09** | 11.43 | 21.1 | 33.9 | 59.2 | **A09** | 4.105 | 15.6 | 15.9 | 51.8 | **A09** | 1.265 | 7.4 | 7.81 | 9.96 | **A09** | 0.705 | 1.62 | 2.43 | 14.1 |
| **Mean** | 8.93 | 19.96 | 25.84 | 59.40 | **Mean** | 7.92 | 17.86 | 20.03 | 47.47 | **Mean** | 1.30 | 4.12 | 4.71 | 10.89 | **Mean** | 1.23 | 3.18 | 4.02 | 14.13 |
| **T CD4+HLA-DR+** | | | | | **T CD8+HLA-DR+** | | | | | **T CD4-CD8-HLA-DR+** | | | | | **T γδ+HLA-DR+** | | | | |
|  | **CN** | **BCG** | **Mtb** | **PHA** |  | **CN** | **BCG** | **Mtb** | **PHA** |  | **CN** | **BCG** | **Mtb** | **PHA** |  | **CN** | **BCG** | **Mtb** | **PHA** |
| **A07** | 28.95 | 65.8 | 67 | x | **A07** | 29.55 | 66.2 | 62.2 | x | **A07** | 15.3 | 23.7 | 20.5 | X | **A07** | 36.65 | 60 | 64,8 | x |
| **A01** | 4.62 | 8.35 | 6.93 | x | **A01** | 13.15 | 34.5 | 23.6 | x | **A01** | 2.51 | 4.88 | 4.91 | X | **A01** | 9.35 | 17.8 | 24,9 | x |
| **A02** | 3.22 | 5.3 | 49.5 | x | **A02** | 10.5 | 19.5 | 21.4 | x | **A02** | 10.8 | 29.3 | 33.6 | X | **A02** | 5.65 | 33.3 | 37,2 | x |
| **A04** | 17.4 | 369 | 47.3 | x | **A04** | 10.75 | 23.1 | 42.3 | x | **A04** | 13 | 28.8 | 28.9 | X | **A04** | 15 | 29.9 | 33,4 | x |
| **A06** | 11.85 | 22.8 | 39 | 56.7 | **A06** | 4.81 | 9.8 | 13.6 | 41 | **A06** | 6.1 | 15.7 | 20.9 | 38.8 | **A06** | 18.05 | 46 | 55,1 | 65 |
| **A08** | 15.2 | 29.2 | 36.4 | 55.9 | **A08** | 4.88 | 6.38 | 10.6 | 50.3 | **A08** | 2.725 | 5.71 | 6.9 | 34.4 | **A08** | 9.2 | 25.3 | 30,9 | 63.7 |
| **A09** | 2.7 | 11 | 36.1 | 58.9 | **A09** | 0.6 | 5.71 | 13 | 56.3 | **A09** | 1 | 1.9 | 2.52 | 34.9 | **A09** | 2.6 | 13 | 16.2 | 68.4 |
| **Mean** | 11.99 | 25.62 | 40.32 | 57.17 | **Mean** | 10.61 | 23.60 | 26.67 | 49.20 | **Mean** | 7.35 | 15.71 | 16.89 | 36.03 | **Mean** | 13.79 | 32.19 | 37.5 | 65.7 |
| **T CD4+CD45RA+** | | | | | **T CD4+CD45RO+** | | | | | **T CD4+CD45RO+CCR7+** | | | | | **T CD4+CD45RO+CCR7-** | | | | |
|  | **CN** | **BCG** | **Mtb** | **PHA** |  | **CN** | **BCG** | **Mtb** | **PHA** |  | **CN** | **BCG** | **Mtb** | **PHA** |  | **CN** | **BCG** | **Mtb** | **PHA** |
| **A01** | 19.95 | 18.9 | 17.4 | x | **A01** | 12 | 17 | 29.4 | x | **A01** | 10.8 | 18.4 | 28.2 | x | **A01** | 89.2 | 81.6 | 71.8 | X |
| **A02** | 19.1 | 18.2 | 15.2 | x | **A02** | 8.7 | 14.3 | 21.2 | x | **A02** | 9.5 | 16.1 | 27.1 | x | **A02** | 90.6 | 83.9 | 72.9 | X |
| **A04** | 22.3 | 33.5 | 19.4 | x | **A04** | 2.1 | 17.2 | 25.1 | x | **A04** | 9 | 25.7 | 32,2 | x | **A04** | 91 | 74.3 | 67.8 | X |
| **A06** | 23 | 23 | 23.4 | 49 | **A06** | 14.4 | 11.3 | 27.1 | 42.7 | **A06** | 8.5 | 22.3 | 34 | 41.1 | **A06** | 91.5 | 77.7 | 66 | 68.9 |

**Supplementary Table 3 (cont.).** Frequency values from Flow Cytometry analyses.

|  | **T CD4+CD45RA+** | | | |  | **T CD4+CD45RO+** | | | |  | **T CD4+CD45RO+CCR7+** | | | |  | **T CD4+CD45RO+CCR7-** | | | |
| --- | --- | --- | --- | --- | --- | --- | --- | --- | --- | --- | --- | --- | --- | --- | --- | --- | --- | --- | --- |
|  | **CN** | **BCG** | **Mtb** | **PHA** |  | **CN** | **BCG** | **Mtb** | **PHA** |  | **CN** | **BCG** | **Mtb** | **PHA** |  | **CN** | **BCG** | **Mtb** | **PHA** |
| **A08** | 21.1 | 20.9 | 21.6 | 51.3 | **A08** | 15.4 | 16.3 | 26.1 | 45.8 | **A08** | 9.6 | 26.6 | 25.8 | 43.3 | **A08** | 90.4 | 73.4 | 74.2 | 66.7 |
| **A09** | 21.8 | 21.5 | 23.3 | 44.5 | **A09** | 16.5 | 8 | 29.5 | 45.3 | **A09** | 5.2 | 22.2 | 29.7 | 48.3 | **A09** | 94.8 | 77.8 | 70.3 | 61.7 |
| **Mean** | 21.18 | 22.67 | 20.05 | 48.27 | **Mean** | 11.5 | 14.02 | 26.4 | 44.6 | **Mean** | 8.76 | 21.88 | 29.51 | 44.23 | **Mean** | 91.24 | 78.12 | 70.5 | 65.77 |
| **T CD8+CD45RA+** | | | | | **T CD8+CD45RO+** | | | | | **T CD8+CD45RO+CCR7+** | | | | | **T CD8+CD45RO+CCR7-** | | | | |
|  | **CN** | **BCG** | **Mtb** | **PHA** |  | **CN** | **BCG** | **Mtb** | **PHA** |  | **CN** | **BCG** | **Mtb** | **PHA** |  | **CN** | **BCG** | **Mtb** | **PHA** |
| **A01** | 59.4 | 50.7 | 59 | x | **A01** | 8.6 | 14 | 12.6 | x | **A01** | 17.3 | 28.3 | 27.1 | x | **A01** | 82.7 | 71.7 | 72.9 | x |
| **A02** | 57.9 | 58.3 | 57 | x | **A02** | 4.1 | 12.1 | 15.3 | x | **A02** | 15.8 | 31.2 | 25.3 | x | **A02** | 84.2 | 68.8 | 74.7 | x |
| **A04** | 45.6 | 60.2 | 61.1 | x | **A04** | 4.8 | 11.5 | 21 | x | **A04** | 15 | 20 | 22.5 | x | **A04** | 85 | 80 | 77.5 | x |
| **A06** | 54.0 | 56.1 | 55 | 72.3 | **A06** | 3.0 | 11.6 | 16 | 25.6 | **A06** | 15.5 | 24.7 | 26.8 | 70.8 | **A06** | 84.5 | 75.3 | 73.2 | 29.2 |
| **A08** | 45.4 | 60.7 | 59.6 | 77 | **A08** | 2.6 | 13.1 | 13.9 | 28.4 | **A08** | 17.9 | 23.8 | 30.7 | 67.5 | **A08** | 82.1 | 76.2 | 69.3 | 32.5 |
| **A09** | 52.1 | 51.9 | 63.4 | 74.6 | **A09** | 12.0 | 16.3 | 17 | 22.3 | **A09** | 17.3 | 26.9 | 28.6 | 73.3 | **A09** | 82.7 | 73.1 | 71.4 | 26.7 |
| **Mean** | 52.38 | 56.32 | 59.18 | 74.63 | **Mean** | 5.85 | 13.09 | 15.97 | 25.43 | **Mean** | 16.47 | 25.82 | 26.84 | 70.53 | **Mean** | 83.53 | 74.18 | 73.16 | 29.47 |
