# Supplementary material for: Impaired macrophage and memory T-cell responses to Bacillus Calmette-Guerin nonpolar lipid extract
Source: Front Immunol. 2024 Jan 11;14:1263352. doi: 10.3389/fimmu.2023.1263352 (PMC10808680; doi:10.3389/fimmu.2023.1263352)
Supplement: Supplementary file 5 [file Table_4.docx]

**Supplementary Table 4.** Median Fluorescence Intensity (MFI) values from Flow Cytometry analyses.

| **T CD4+TNF+** | | | | | **T CD4+IFN+** | | | | | **T CD4+IL-2+** | | | | | **T CD4+IL-17+** | | | | |
| --- | --- | --- | --- | --- | --- | --- | --- | --- | --- | --- | --- | --- | --- | --- | --- | --- | --- | --- | --- |
|  | **CN** | **BCG** | **Mtb** | **PHA** |  | **CN** | **BCG** | **Mtb** | **PHA** |  | **CN** | **BCG** | **Mtb** | **PHA** |  | **CN** | **BCG** | **Mtb** | **PHA** |
| **A01** | 318 | 1295 | 2248 | x | **A01** | 613 | 1129 | 1660 | x | **A01** | 559 | 1124 | 1121 | x | **A01** | 1111 | 2166 | 2194 | x |
| **A02** | 429 | 1181 | 3218 | x | **A02** | 688 | 1388 | 2275 | x | **A02** | 507 | 1034 | 1017 | x | **A02** | 1919 | 2054 | 1983 | x |
| **A04** | 462 | 1353 | 2152 | x | **A04** | 537 | 1210 | 1109 | x | **A04** | 536 | 1066 | 1058 | x | **A04** | 1589 | 6387 | 5438 | x |
| **A06** | 474 | 1017 | 1017 | 13352 | **A06** | 794 | 1296 | 1470 | 18019 | **A06** | 111 | 1375 | 3289 | 12715 | **A06** | 1114 | 5350 | 6248 | 37930 |
| **A08** | 546 | 1039 | 1297 | 13391 | **A08** | 615 | 1361 | 1565 | 17582 | **A08** | 120 | 1204 | 1580 | 12040 | **A08** | 1849 | 3374 | 3732 | 37432 |
| **A09** | 303 | 1326 | 2379 | 13960 | **A09** | 861 | 1189 | 2669 | 14101 | **A09** | 181 | 1420 | 2020 | 14401 | **A09** | 1871 | 4020 | 3364 | 29975 |
| **T CD8+TNF+** | | | | | **T CD8+IFN+** | | | | | **T CD8+IL-2+** | | | | | **T CD8+IL-17+** | | | | |
|  | **CN** | **BCG** | **Mtb** | **PHA** |  | **CN** | **BCG** | **Mtb** | **PHA** |  | **CN** | **BCG** | **Mtb** | **PHA** |  | **CN** | **BCG** | **Mtb** | **PHA** |
| **A01** | 735 | 1529 | 2116 | x | **A01** | 521 | 1674 | 1226 | x | **A01** | 417 | 809 | 820 | x | **A01** | 1019 | 1964 | 1993 | x |
| **A02** | 342 | 1115 | 2204 | x | **A02** | 613 | 4129 | 5907 | x | **A02** | 554 | 1118 | 1106 | x | **A02** | 1306 | 2107 | 2029 | x |
| **A04** | 579 | 1104 | 2370 | x | **A04** | 524 | 2802 | 3756 | x | **A04** | 549 | 1130 | 1118 | x | **A04** | 1166 | 4329 | 4189 | x |
| **A06** | 818 | 1250 | 1707 | 11688 | **A06** | 887 | 1867 | 1333 | 15986 | **A06** | 1383 | 3026 | 5935 | 37651 | **A06** | 1623 | 2243 | 1953 | 17994 |
| **A08** | 636 | 1119 | 2434 | 13393 | **A08** | 530 | 1861 | 1727 | 14697 | **A08** | 625 | 1647 | 2545 | 22760 | **A08** | 732 | 1558 | 1732 | 16520 |
| **A09** | 300 | 1669 | 1266 | 14012 | **A09** | 596 | 1647 | 3198 | 13653 | **A09** | 1990 | 3966 | 2075 | 24583 | **A09** | 1026 | 1652 | 1483 | 15402 |
| **T CD4-CD8-TNF+** | | | | | **T CD4-CD8-IFN+** | | | | | **T CD4-CD8-IL-2+** | | | | | **T CD4-CD8-IL-17+** | | | | |
|  | **CN** | **BCG** | **Mtb** | **PHA** |  | **CN** | **BCG** | **Mtb** | **PHA** |  | **CN** | **BCG** | **Mtb** | **PHA** |  | **CN** | **BCG** | **Mtb** | **PHA** |
| **A01** | 351 | 1117 | 1201 | x | **A01** | 686 | 1147 | 1345 | x | **A01** | 586 | 1115 | 1172 | x | **A01** | 741 | 1441 | 1489 | x |
| **A02** | 372 | 1129 | 1568 | x | **A02** | 648 | 1164 | 1357 | x | **A02** | 673 | 1223 | 1115 | x | **A02** | 855 | 1504 | 1522 | x |
| **A04** | 372 | 1138 | 1337 | x | **A04** | 624 | 1266 | 1349 | x | **A04** | 632 | 1189 | 1109 | x | **A04** | 863 | 1585 | 1529 | x |
| **A06** | 390 | 1133 | 1322 | 12470 | **A06** | 608 | 1264 | 1479 | 11221 | **A06** | 1048 | 5403 | 8761 | 26070 | **A06** | 562 | 3394 | 3394 | 19470 |
| **A08** | 420 | 1158 | 1306 | 11245 | **A08** | 667 | 1293 | 1367 | 11545 | **A08** | 1986 | 5125 | 3650 | 25640 | **A08** | 1461 | 1623 | 9494 | 15446 |
| **A09** | 489 | 1163 | 1366 | 12132 | **A09** | 573 | 1210 | 1261 | 11571 | **A09** | 1407 | 5093 | 2598 | 30808 | **A09** | 1258 | 1026 | 9005 | 19243 |
