# Supplementary material for: Impaired macrophage and memory T-cell responses to Bacillus Calmette-Guerin nonpolar lipid extract
Source: Front Immunol. 2024 Jan 11;14:1263352. doi: 10.3389/fimmu.2023.1263352 (PMC10808680; doi:10.3389/fimmu.2023.1263352)
Supplement: Supplementary file 6 [file Table_5.docx]

**Supplementary Table 5.** Concentration (pg/mL) levels of IFNγ and IL-10 from ELISA analyses.

| **IFNγ** | **24 h (Lipids)** | **Samples** | **pg/mL** | **48 h (Lipids)** | **Samples** | **pg/mL** | **72 h (Lipids)** | **Samples** | **pg/mL** |
| --- | --- | --- | --- | --- | --- | --- | --- | --- | --- |
|  |  | CNA06 | 19.88 |  | CNA01 | 5.58 |  | CNA04 | 105.46 |
|  |  | CNA15 | 34.01 |  | CNA02 | 14.31 |  | CNA05 | 105.06 |
|  |  | CNA17 | 103.49 |  | CNA03 | 14.11 |  | CNA06 | 114.48 |
|  |  | BCGA06 | 52.85 |  | CNA04 | 3.15 |  | BCGA04 | 46.18 |
|  |  | BCGA15 | 134.11 |  | CNA06 | 12.08 |  | BCGA05 | 201.24 |
|  |  | BCGA17 | 138.43 |  | CNA07 | 13.50 |  | BCGA06 | 161.20 |
|  |  | MtbA06 | 288.77 |  | CNA08 | 11.88 |  | MtbA04 | 228.32 |
|  |  | MtbA15 | 278.57 |  | CNA09 | 11.07 |  | MtbA05 | 223.61 |
|  |  | MtbA17 | 222.04 |  | BCGA01 | 179.75 |  | MtbA06 | 222.04 |
|  |  | PHAA06 | 1,159.83 |  | BCGA02 | 122.92 |  | PHAA04 | 713.11 |
|  |  | PHAA15 | 1,560.62 |  | BCGA03 | 161.28 |  | PHAA05 | 623.61 |
|  |  | PHAA17 | 1,182.20 |  | BCGA04 | 140.37 |  | PHAA06 | 642.46 |
|  |  | - | - |  | BCGA06 | 103.22 |  | - | - |
|  |  | - | - |  | BCGA07 | 104.85 |  | - | - |
|  |  | - | - |  | BCGA08 | 104.04 |  | - | - |
|  |  | - | - |  | BCGA09 | 101.60 |  | - | - |
|  |  | - | - |  | MtbA01 | 281.25 |  | - | - |
|  |  | - | - |  | MtbA02 | 367.73 |  | - | - |
|  |  | - | - |  | MtbA03 | 219.95 |  | - | - |
|  |  | - | - |  | MtbA04 | 258.72 |  | - | - |
|  |  | - | - |  | MtbA06 | 336.47 |  | - | - |
|  |  | - | - |  | MtbA07 | 340.53 |  | - | - |
|  |  | - | - |  | MtbA08 | 313.73 |  | - | - |
|  |  | - | - |  | MtbA09 | 315.96 |  | - | - |

**Supplementary Table 5 (cont.).** Concentration (pg/mL) levels of IFNγ and IL-10 from ELISA analyses.

| **IFNγ** | **24h (Lipids)** | **Samples** | **pg/mL** | **48h (Lipids)** | **Samples** | **pg/mL** | **72h (Lipids)** | **Samples** | **pg/mL** |
| --- | --- | --- | --- | --- | --- | --- | --- | --- | --- |
|  |  | - | - |  | PHAA06 | 529.31 |  | - | - |
|  |  | - | - |  | PHAA08 | 544.53 |  | - | - |
|  |  | - | - |  | PHAA09 | 531.75 |  | - | - |
| **IL-10** | **24h (Lipids)** | **Samples** | **pg/mL** | **48h (Lipids)** | **Samples** | **pg/mL** | **72h (Lipids)** | **Samples** | **pg/mL** |
|  |  | CNA06 | 38.37 |  | CNA01 | 25.41 |  | CNA04 | 2.86 |
|  |  | CNA15 | 70.93 |  | CNA02 | 26.46 |  | CNA05 | 12.99 |
|  |  | CNA17 | 25.76 |  | CNA03 | 14.56 |  | CNA06 | 13.41 |
|  |  | BCGA06 | 56.92 |  | CNA04 | 4.76 |  | BCGA04 | 136.69 |
|  |  | BCGA15 | 199.42 |  | CNA05 | 27.51 |  | BCGA05 | 108.83 |
|  |  | BCGA17 | 243.88 |  | CNA06 | 26.11 |  | BCGA06 | 113.68 |
|  |  | MtbA06 | 412.99 |  | CNA07 | 10.71 |  | MTBA04 | 169.41 |
|  |  | MtbA15 | 589.09 |  | CNA08 | 0.91 |  | MTBA05 | 127.62 |
|  |  | MtbA17 | 339.46 |  | CNA09 | 0.91 |  | MTBA06 | 147.25 |
|  |  | PHAA06 | 1,215.09 |  | BCGA01 | 257.19 |  | PHAA04 | 206.99 |
|  |  | PHAA15 | 1,378.94 |  | BCGA02 | 255.79 |  | PHAA05 | 202.35 |
|  |  | PHAA17 | 1,042.84 |  | BCGA03 | 174.91 |  | PHAA06 | 205.30 |
|  |  | - | - |  | BCGA04 | 230.58 |  | - | - |
|  |  | - | - |  | BCGA05 | 266.99 |  | - | - |
|  |  | - | - |  | BCGA06 | 248.08 |  | - | - |
|  |  | - | - |  | BCGA07 | 88.43 |  | - | - |
|  |  | - | - |  | BCGA08 | 18.06 |  | - | - |
|  |  | - | - |  | BCGA09 | 2.31 |  | - | - |

**Supplementary Table 5 (cont.).** Concentration (pg/mL) levels of IFNγ and IL-10 from ELISA analyses.

| **IL-10** | **24h (Lipids)** | **Samples** | **pg/mL** | **48h (Lipids)** | **Samples** | **pg/mL** | **72h (Lipids)** | **Samples** | **pg/mL** |
| --- | --- | --- | --- | --- | --- | --- | --- | --- | --- |
|  |  | - | - |  | MtbA01 | 264.19 |  | - | - |
|  |  | - | - |  | MtbA02 | 255.44 |  | - | - |
|  |  | - | - |  | MtbA03 | 181.91 |  | - | - |
|  |  | - | - |  | MtbA04 | 257.54 |  | - | - |
|  |  | - | - |  | MtbA05 | 258.94 |  | - | - |
|  |  | - | - |  | MtbA06 | 250.54 |  | - | - |
|  |  | - | - |  | MtbA07 | 85.28 |  | - | - |
|  |  | - | - |  | MtbA08 | 286.25 |  | - | - |
|  |  | - | - |  | MtbA09 | 173.51 |  | - | - |
|  |  | - | - |  | PHAA06 | 341.56 |  | - | - |
|  |  | - | - |  | PHAA08 | 358.37 |  | - | - |
|  |  | - | - |  | PHAA09 | 365.72 |  | - | - |
