# Supplementary material for: Impaired macrophage and memory T-cell responses to Bacillus Calmette-Guerin nonpolar lipid extract
Source: Front Immunol. 2024 Jan 11;14:1263352. doi: 10.3389/fimmu.2023.1263352 (PMC10808680; doi:10.3389/fimmu.2023.1263352)
Supplement: Supplementary file 7 [file Table_6.docx]

**Supplementary Table 6.** List of flow cytometry antibodies.

| **Antibody** | **Clone** | **Material Number** | **Company** |
| --- | --- | --- | --- |
| FITC Mouse Anti-Human CD3 | UCHT1 | 300406 | BD Bioscience |
| PE Mouse Anti-Human CD4 | OKT4 | 317410 | BD Bioscience |
| APC-Cy™7 Mouse Anti-Human CD8 | SK1 | 557834 | BD Bioscience |
| PE-Cy™7 Mouse Anti-Human CD45RA | HI100 | 560675 | BD Bioscience |
| APC Mouse Anti-Human CD45RO | UHCL1 | 559865 | BD Bioscience |
| BV510 Mouse Anti-Human CCR7 | 2-L1-A | 566760 | BD Bioscience |
| BV605 Mouse Anti-Human HLA-DR | G46-6 | 562845 | BD Bioscience |
| BV421 Mouse Anti-Human TCRγσ | BI | 562560 | BD Bioscience |
| PE-Cy™7 Mouse Anti-Human IFNγ | B27 | 557643 | BD Bioscience |
| Alexa Fluor® 700 Mouse Anti-Human TNF | Mab11 | 557996 | BD Bioscience |
| BV421 Mouse Anti-Human IL-2 | MQ1-17H12 | 564164 | BD Bioscience |
| BV510 Mouse Anti-Human IL-17A | N49-653 | 563295 | BD Bioscience |
